# Improving hand hygiene compliance in Singapore via innovation

**DOI:** 10.1186/1753-6561-5-S6-P124

**Published:** 2011-06-29

**Authors:** L Ang, KL Ooi, A Lim, B Ong, P Tambyah, D Fisher

**Affiliations:** 1 National University Hospital, Singapore, Singapore

## Introduction / objectives

The National University Hospital (NUH) is a 997 bed acute tertiary hospital. In 2006, we embarked on a MRSA prevention bundle, the cornerstone of which was a hand hygiene (HH) compliance programme.

## Methods

HH is a standard for NUH hospital accreditation. In 2007 it became an internal key performance area with alcohol hand rub fitted to the foot of all beds. HH audits, using the WHO toolkit were conducted by Infection Control Liaison Nurses across hospitals, medical students and staff from our partner health cluster. Ward-specific audit results were displayed publicly in wards. Poor results were reported to nominated senior clinical ’HH Champions‘. Good compliance results were recognised by the senior leadership. In 2009, a one week training programme demonstrated the WHO 5 moments for HH and the 6 steps to 4000 staff. HH competence was determined with UV lights and “glogerm”. Staff signed a contract and pledged to compliance. Movement activated audio reminders were placedÂ onÂ 3 wards along with posters, hospital wide. On May 5, 2010, a media campaign was launched with a 13 metre high sign posted at the NUH’s façade and internal events including awards and a roaming hand mascot.

Medical and nursing students undertake compulsory HH audits. Candidates in the final medical student (MBBS) clinical examination lose marks for failed compliance.

## Results

We published HH compliance rates of 16% in 2006. Through 2010, our rate was 64% with about 1000 observed opportunities/month. Our hand rub purchases increased significantly from 391 units (sd 87) in 2006 to 1760 (sd 308) in 2010.

## Conclusion

HH compliance requires active commitment from all, particularly top management. Formal and informal education, stringent audits, public displays and senior management support has significantly raised the HH compliance in NUH.

## Disclosure of interest

None declared.

